# Compression-Induced Phase Transitions of Bicalutamide

**DOI:** 10.3390/pharmaceutics12050438

**Published:** 2020-05-09

**Authors:** Joanna Szafraniec-Szczęsny, Agata Antosik-Rogóż, Justyna Knapik-Kowalczuk, Mateusz Kurek, Ewa Szefer, Karolina Gawlak, Krzysztof Chmiel, Sebastian Peralta, Krzysztof Niwiński, Krzysztof Pielichowski, Marian Paluch, Renata Jachowicz

**Affiliations:** 1 Department of Pharmaceutical Technology and Biopharmaceutics, Faculty of Pharmacy, Jagiellonian University Medical College, Medyczna 9, 30-688 Krakow, Poland; agata.antosik@uj.edu.pl (A.A.-R.); Mateusz.kurek@uj.edu.pl (M.K.); Krzysztof.niwinski@uj.edu.pl (K.N.); renata.jachowicz@uj.edu.pl (R.J.); 2 Department of Physical Chemistry and Electrochemistry, Faculty of Chemistry, Jagiellonian University, Gronostajowa 2, 30-387 Krakow, Poland; gawlak@chemia.uj.edu.pl; 3 Faculty of Science and Technology, Institute of Physics, University of Silesia, SMCEBI, 75 Pułku Piechoty 1a, 41-500 Chorzów, Poland; justyna.knapik-kowalczuk@smcebi.edu.pl (J.K.-K.); krzysztof.chmiel@smcebi.edu.pl (K.C.); marian.paluch@us.edu.pl (M.P.); 4 Department of Chemistry and Technology of Polymers, Cracow University of Technology, Warszawska 24, 31-155 Krakow, Poland; ewa.szefer@doktorant.pk.edu.pl (E.S.); kpielich@pk.edu.pl (K.P.); 5 Pharmacy and Pharmaceutical Technology Department, School of Pharmacy, University of Granada, Campus de Cartuja s/n., 18071 Granada, Spain; seperalta@ugr.es

**Keywords:** amorphous solid dispersions, physical stability, bicalutamide, Kollidon^®^VA64, dissolution, compression

## Abstract

The formation of solid dispersions with the amorphous drug dispersed in the polymeric matrix improves the dissolution characteristics of poorly soluble drugs. Although they provide an improved absorption after oral administration, the recrystallization, which can occur upon absorption of moisture or during solidification and other formulation stages, serves as a major challenge. This work aims at understanding the amorphization-recrystallization changes of bicalutamide. Amorphous solid dispersions with poly(vinylpyrrolidone-*co*-vinyl acetate) (PVP/VA) were obtained by either ball milling or spray drying. The applied processes led to drug amorphization as confirmed using X-ray diffraction and differential scanning calorimetry. Due to a high propensity towards mechanical activation, the changes of the crystal structure of physical blends of active pharmaceutical ingredient (API) and polymer upon pressure were also examined. The compression led to drug amorphization or transition from form I to form II polymorph, depending on the composition and applied force. The formation of hydrogen bonds confirmed using infrared spectroscopy and high miscibility of drug and polymer determined using non-isothermal dielectric measurements contributed to the high stability of amorphous solid dispersions. They exhibited improved wettability and dissolution enhanced by 2.5- to 11-fold in comparison with the crystalline drug. The drug remained amorphous upon compression when the content of PVP/VA in solid dispersions exceeded 20% or 33%, in the case of spray-dried and milled systems, respectively.

## 1. Introduction

The issue of solubility of active pharmaceutical ingredients (APIs) is a matter of concern during drug development. The number of poorly soluble APIs exceeds 50% of currently marketed drugs and 70% of newly synthesized chemical entities [1]. A formation of amorphous solid dispersions allows for overcoming the poor dissolution of crystalline APIs in solid dosage forms. It is due to the high levels of supersaturation in solution after oral administration [2]. However, the lack of long-range order of crystal lattice leads to the instability of amorphous substances manifested by a tendency towards recrystallization. Given that drug compound in solid dispersion must remain amorphous to maintain its beneficial dissolution characteristics, recrystallization can undermine this favorable effect. To overcome this drawback, APIs are combined with water-soluble polymers that raise the overall glass transition temperature (*T_g_*) of the solid dispersion. The anti-plasticizing effect leads to a reduction of molecular mobility, which is essential for maintaining the physical stability of the amorphous drug substances [3].

The use of hydrophilic carriers such as polyvinylpyrrolidone (PVP) or poly(vinylpyrrolidone-*co*-vinyl acetate) (PVP/VA) enhances the wettability of the system. It leads to an increase in the dissolution of API in comparison to its crystalline counterpart. However, those hygroscopic polymers may absorb water when exposed to the humidity, which reduces the physical stability of pharmaceutical compositions [4]. On the other hand, the high values of *T_g_* and high API solubilizing ability of PVP-based polymers means they are frequently used in formulation studies. Although the solid solubility of a drug in polymeric matrices in ambient conditions is low, usually not exceeding 30%, the use of PVP and its derivatives provide relatively high miscibility with drug substances. Moreover, in many cases, even a small amount of those polymers can stabilize the amorphous form of numerous APIs, as described for felodipine or acetaminophen [5,6]. It results from the formation of strong intermolecular interactions, such as hydrogen bonds, that influence the nucleation and crystal growth [7].

The issue of recrystallization upon storage is one of the main challenges that amorphous solid dispersions need to face to be introduced into the market. Hence, the effect of humidity and temperature on physical stability has been widely studied. However, several issues must also be considered in the research and development process. One of them is recrystallization during solidification and further formulation stages [8]. Technological processes such as tableting may induce phase separation, nucleation, and facilitate the crystallization of amorphous systems, as described for indomethacin, itraconazole, naproxen, and nimodipine [9,10,11,12]. The studies performed so far showed that the compression-induced crystallization could be a severe limitation in the formulation studies of API exhibiting relatively high stability and low recrystallization tendency of the amorphous forms, as shown for chloramphenicol and etoricoxib [13,14]. On the other hand, it was also shown that the addition of PVP-based copolymers improves the stability upon compression of naproxen, flutamide, and sucrose, among others [15,16,17].

Bicalutamide is a nonsteroidal drug that blocks the growth-simulating effect of androgens on the prostate tumor, the fourth most diagnosed worldwide [18,19]. It is a lipophilic compound (logP = 2.92) of low water solubility (3.7 mg/L), assigned to class II of the Biopharmaceutics Classification System [20]. The conformational flexibility of the bicalutamide molecule means it exhibits polymorphism and reduced crystallization propensity [21,22]. Given that the polymorph transitions affect the physicochemical properties of the drug, the studies of the relationship between the structure and the properties has become essential, with the emphasis put on the drug dissolution characteristics. The papers published so far have shown that the drug easily undergoes amorphization, which leads to an increase in its dissolution [23,24]. The inhibition of crystallization was found to be a function of polymer concentration. However, the stability issue upon compression has not yet been investigated.

In the paper presented herein, we investigated the effect of the stabilization of amorphous bicalutamide by PVP/VA copolymer present in low concentrations. Either ball milling or spray drying was applied to obtain solid dispersions containing 50%, 66%, 80%, or 90% of the drug. The processes led to changes in particle size and morphology, as confirmed by scanning electron microscopy (SEM) and laser diffraction studies. The results of X-ray diffraction and differential scanning calorimetry indicated that block copolymer of vinylpyrrolidone and vinyl acetate (Kollidon^®^VA64) stabilized the amorphous bicalutamide. The improvement in drug dissolution from solid dispersions was observed as well. Infrared spectroscopy was applied to follow the interactions between drug and polymer molecules, and contact angle measurements were performed to access the wettability of solid dispersions. Additionally, we evaluated the effect of compression on the molecular arrangement of either: (i) crystalline bicalutamide (form I polymorph) physically mixed with the polymer, or (ii) amorphous drug in solid dispersions. Given that the advantageous solubility improvement of high-energy solids is often reduced by the physical instability and the propensity towards recrystallization, this issue is particularly important from the perspective of pharmaceutical technology.

## 2. Materials and Methods

### 2.1. Materials

Bicalutamide (BCL, *N*-[4-cyano-3-(trifluoromethyl)phenyl]-3-[(4-fluorophenyl) sulfonyl]-2-hydroxy-2-methylpropanamide, 99.8%, Hangzhou Hyper Chemicals Limited, Hangzhou, China) was used as a model drug. Vinylpyrrolidone-vinyl acetate copolymer (Kollidon^®^VA64, PVP/VA, BASF, Ludwigshafen am Rhein, Germany) was used as an excipient. Sodium lauryl sulfate (SLS, BASF, Ludwigshafen am Rhein, Germany) was used as a surfactant in the preparation of the dissolution medium. Ethanol (absolute, 99.8%, pure p.a., Avantor Performance Materials, Gliwice, Poland) was used as a solvent in spray drying processes. Cyclohexane (ACS, pure p.a., Avantor Performance Materials, Gliwice, Poland) was used as a dispersant in laser diffraction measurements. All chemicals were used as received. Distilled water was used to prepare all the aqueous solutions.

### 2.2. Spray Drying

A Büchi Mini Spray Dryer B-191 (Flawil, Switzerland) was used to obtain solid dispersions containing bicalutamide and PVP/VA (BCL-PVP/VA). The ethanolic solutions containing drug and polymer (1:1, 2:1, 4:1, and 10:1 wt. ratio, respectively) were spray-dried using the following parameters: inlet temperature: 76 °C, outlet temperature: 58 °C, aspirator flow: 100%, gas flow rate: 600 L/min, liquid flow rate: 3.4 mL/min, 0.7 mm in diameter single-fluid nozzle, yields ca. 70%. The process was carried out under constant control, and the concentration of ethanol was 10-times lower than the flammability limit. The samples were further dried under vacuum to remove residual solvent.

### 2.3. Planetary Ball Milling

A high-energy planetary ball mill Pulverisette 7 (Fritsch, Idar-Oberstein, Germany) was used to obtain solid dispersions. Crystalline bicalutamide (form I polymorph) was mixed with PVP/VA in 1:1, 2:1, 4:1, and 10:1 w/w ratios respectively, and milled at room temperature in the zirconium oxide milling jars (45 mL) filled with seven zirconium balls (15 mm in diameter) rotating at 400 rpm. A grinding cycle was divided into a 20 min milling process and a 10 min pause and repeated 35 times. A reverse mode was used to avoid an overheating of the samples.

### 2.4. Scanning Electron Microscopy (SEM)

A Phenom Pro desktop electron microscope (PhenomWorld, Thermo Fisher Scientific, Waltham, MA, USA) equipped with a CeB_6_ electron source and backscattered electron detector was used to determine the size and morphology of the samples. The acceleration voltage was equal to 10 kV. The powder was placed on the conductive adhesive tape previously glued to the specimen mount. The excess of the sample (loosely bound to the tape) was removed using a stream of argon. The holder for non-conductive samples was used.

### 2.5. Differential Scanning Calorimetry (DSC)

The thermal properties of the investigated mixtures were examined using a DSC 1 STAR^e^ System (Mettler–Toledo, Greifensee, Switzerland). The measuring device was equipped with an HSS8 ceramic sensor having 120 thermocouples and a liquid nitrogen cooling station. The instrument was calibrated for temperature and enthalpy using zinc and indium standards. The glass transition temperature, crystallization, and melting were determined as the midpoint of the glass transition step, the onset of the exothermic peak, and the peak of the endothermic event, respectively. The samples were measured in an aluminum crucible (40 μL). The sample mass used for DSC experiments varied between 7 to 10 mg. All measurements were carried out with and without annealing (T = 323 K; t = 10 min). The experiments were performed from 273 to 478 K, with a heating rate equal to 10 K/min.

### 2.6. Powder X-ray Diffraction (PXRD)

An X-ray diffractometer Rigaku Mini Flex II (Tokyo, Japan) was used to register the diffraction patterns of the samples. The angular range 3–70° 2θ was scanned in steps of 0.02 with a scan speed equal to 5°/min. The measurements were carried out at ambient temperature using monochromatic Cu Kα radiation (λ = 1.5418 Å). The samples in the form of powder or tablets were placed in a standard glass sample holder without milling before the measurement.

### 2.7. Fourier Transform Infrared Spectroscopy (FTIR)

The vibrational spectra of solid dispersions were collected using a Nicolet iS10 FT-IR spectrometer (Thermo Fisher Scientific, Waltham, MA, USA) equipped with a Smart iTR™ ATR (Attenuated Total Reflectance) sampling accessory with a diamond as an ATR crystal. Spectra were collected within the range between 600 and 4000 cm^−1^ with 8 cm^−1^ resolution and presented as the averages from 128 scans collected for each sample.

### 2.8. Laser Diffraction Measurements

A wet method of determination of particle size distribution was performed using a Malvern Mastersizer 3000 equipped with a HydroEV unit (Malvern, United Kingdom). Cyclohexane (reflective index, RI = 1.426) was filtered through the G5 sintered disc filter funnel and placed in the beaker. The sample in powder form was added to the dispersant until the obscuration reached the given value (between 5% and 20%), and then the measurement was carried out. The rotational speed of the mixer was 1500 rpm. The relationship between the particle size and light intensity distribution pattern was found based on the Fraunhofer diffraction theory. The reported data represents the averages from six to ten series of measurements of each sample with standard deviations (SD) and the distribution span.

### 2.9. Dissolution Study

A pharmacopeial type 2 (paddle) dissolution apparatus Vision Elite 8 (Hanson Research, Chatsworth, CA, USA) equipped with a VisionG2 AutoPlus Autosampler was used to determine the dissolution of bicalutamide. A method recommended by the U. S. Food and Drug Administration (FDA) for BCL tablets was applied. Briefly, the samples in the form of either powder or compact, an equivalent of 50 mg of BCL, were placed in the dissolution beakers filled with 1000 mL of 1% SLS aqueous solution maintained at 37.0 ± 0.5 °C and mixed at 50 rpm. The amount of dissolved drug was assayed at 272 nm using a Shimadzu UV-1800 spectrophotometer (Shimadzu Corporation, Kyoto, Japan) equipped with flow-through cuvettes. The sink conditions were maintained. The tests were carried out in triplicate, and the presented results represent averages with their standard deviations (Mean ± SD).

### 2.10. Intrinsic Dissolution Rate Study

The compacts for intrinsic dissolution studies were prepared using a Specac 50 hydraulic press (Specac, Kent, UK). The powder systems were compressed directly into the stainless-steel cylinders, having a 0.5026 cm^2^ flat surface area. Two tons of load force was applied for 30 s. The cylinders were then mounted in the Hanson Vision G2 Elite 8 dissolution apparatus (Hanson Research, Chatsworth, CA, USA) and stirred at 100 rpm. The dissolution vessels were filled with 500 mL of 1% SLS solution maintained at 37 °C. The amount of dissolved BCL was assayed online using a UV-1800 spectrophotometer (Shimadzu Corporation, Kioto, Japan) at λ = 272 nm. The intrinsic dissolution rate was calculated based on the accessible surface area as a function of the mass ratio of the components in the mixture. Ten points were used for the construction of intrinsic dissolution profiles of each system, and further to calculate the intrinsic dissolution rate (IDR) values.

### 2.11. Wettability

A DSA255 drop shape analyzer (Krüss, Hamburg, Germany) was used to measure the contact angles. The sessile drop technique was applied. The droplet of distilled water of volume equal to 2 µL was deposited on the surface of solid dispersions compressed using an Atlas^TM^ manual 15 t hydraulic press (Specac, Kent, UK) with a load force of 1.5 t that was applied for each sample for 15 s.

### 2.12. Determination of Water Content

Thermogravimetric analysis (TGA) was performed using a 209F1 Libra thermal analyzer (Netzsch, Selb, Germany), operating in dynamic mode at a heating rate of 10 K/min from 30 to 600 °C under inert (argon) or oxidative (synthetic air) atmosphere. The sample mass was 4.5 mg, and open α-Al_2_O_3_ crucibles were used.

### 2.13. Compression

A weighted amount of prepared solid dispersions was compressed using a single punch eccentric tablet press (Korsch EK0, Berlin, Germany) with the set of circular, flat-faced, 7 mm in diameter punches. The upper punch was equipped with a temperature-compensated, full-bridge, four strain gauges force sensor (CL 21-UJ-T) with measuring range up to 10 kN and a sensitivity of 0.882 mV/V (ZEPWN, Marki, Poland). The sensor signal was collected on PC via an Esam Traveller 1 0508-S converter equipped with an ESAM USB software. During the tableting, the curves of time/compression force relationships were drawn, and the maximal compression force was recorded. To maintain the drug content, each tablet mass (100 mg, 75 mg, 62.5 mg, or 55 mg, respectively) was weighed, put into the die cavity, and tableted manually. Three compression force levels: low, medium, and high were set for each formulation (Table 1). However, the compactibility of some formulations was insufficient to obtain tablets with low and even medium force. As a result, 31 batches of tablets were manufactured.

### 2.14. Broadband Dielectric Spectroscopy (BDS)

The dielectric measurements were carried out using Novo-Control GMBH Alpha dielectric spectrometer (Montabaur, Germany), in the frequency range from 10^−1^ to 10^6^ Hz at given temperatures with a heating rate equal to 0.5 K/min. The temperature was controlled by a Quatro temperature controller with temperature stability better than 0.1 K. Dielectric studies of BCL and its binary systems were performed immediately after its vitrification by fast cooling of the melt in a parallel-plate cell made of stainless steel (diameter 15 mm, and a 0.1 mm gap with quartz spacers).

## 3. Results and Discussion

### 3.1. Size Distribution and Morphological Features of Solid Dispersions

**Figure 1:**
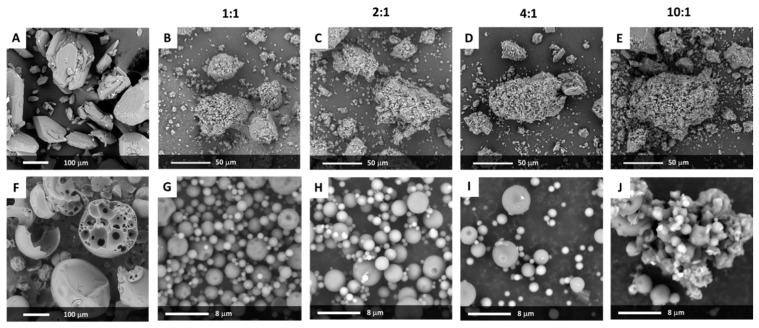
Scanning electron microscopy (SEM) pictures of crystalline bicalutamide (BCL) (**A**), BCL-PVP/VA solid dispersions obtained by either ball milling (**B**–**E**) or spray drying (**G**–**J**), and Kollidon^®^VA64 (**F**). The composition of binary systems is shown above the pictures.

The SEM analysis of crystalline bicalutamide indicates that drug particles exhibited the shape of elongated hexagons with a smooth surface, sharp edges, and the length not exceeding 200 µm along the long axis (Figure 1A). The median of the particle size determined using a laser diffraction technique was equal to 81.7 µm with a span of 1.7. The microscopic image of PVP/VA indicates that the excipient existed in the form of hollow spheres (mostly ruptured) of diameter ranging between 30 and 300 µm, with a small fraction of particles of greater diameter (Figure 1F). The laser diffraction measurements indicated that the median of the particle size distribution of PVP/VA lay at 125.2 µm, and the *D_v_*(90) value was equal to 288.9 µm.

The transfer of mechanical energy during milling led to clearly marked changes in particle morphology. Neither hexagonal crystals of bicalutamide nor the spheres of Kollidon^®^VA64 existed in the sample. The SEM analysis indicated the formation of irregular agglomerates of diameter ranging between 20 and 120 µm, covered by much smaller dust-like particles (Figure 1B–E). Interestingly, no significant differences in particle morphology occurred between the systems containing different amounts of PVP/VA, only the system milled at 10 to 1 wt. ratio formed the particles of the fuzzier surface. It could be a consequence of the recrystallization of bicalutamide and the formation of crystal nuclei on the surface of particles (see further considerations in Section 3.3).

In a spray drying process, the liquid is dispersed in the form of droplets and dried with hot air. It leads to a formation of particles of consistent size distribution, usually spherical or in the shape of ruptured spheres, of diameter less than 10 µm [25]. The SEM analysis confirmed the formation of spherical particles of diameter ranging from hundreds of nanometers to several micrometers (Figure 1G–J). The surface of many particles of the system containing an equal amount of the drug and the carrier was heterogeneous, with visible folds, while the other solid dispersions exhibit smooth particles. Importantly, in the 10:1 system, the particles tended to aggregate and formed structures of less defined geometry and well-visible sharp-edged crystals (Figure 1J).

**Figure 2:**
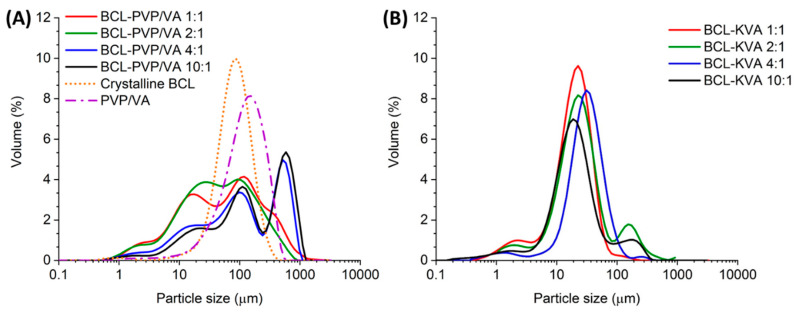
Particle size distribution of BCL-PVP/VA solid dispersions obtained by either ball milling (**A**) or spray drying (**B**).

The distribution of particle size determined using the laser diffraction method indicated that milling led to the formation of particles that differed in size. It agreed with SEM data (Figure 2). The distributions assigned to BCL-PVP/VA 1:1 and 2:1 milled systems are wide and less structured than those determined for 4:1 and 10:1 binary systems. Moreover, when the content of BCL increased, the distribution curves began to exhibit intense and well-resolved maxima originating from the fraction of large particles (Table 2). The *D_v_*(50) value, which is the median for a volume particle distribution, is 2–3 times greater than the one registered for either 1:1 or 2:1 solid dispersion obtained in milling. The inhomogeneous size distribution can be a result of the prolonged transfer of mechanical energy and particle agglomeration.

The size distributions of particles obtained in the spray drying process were narrower than in the case of milled samples, with well visible maxima located in the range between 10 and 100 µm. However, the distributions were not homogenous and tailed within the range of particles not exceeding 10 µm, as well as those greater than 100 µm (Figure 2). In the case of all the systems, the *D_v_*(50) value ranged between 20 and 32 µm, and the span of the distribution was lower than determined for milled samples. The increase in span values calculated for 2:1 and 10:1 spray-dried systems resulted from the observed second maxima above 100 µm. Importantly, the data presented in Figure 2 indicate that each sample contained particles of diameter below 1 µm. It can affect the solubility of the samples according to the Ostwald–Freundlich equation [26].

### 3.2. Thermal Properties

**Figure 3:**
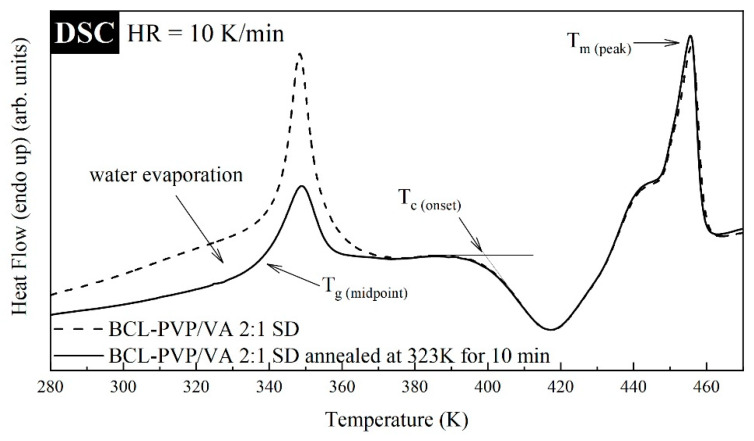
Exemplary differential scanning calorimetry (DSC) thermograms of the BCL-PVP/VA 2:1 spray-dried system before and after annealing.

The thermal properties of BCL-PVP/VA solid dispersions obtained by either spray-drying or ball milling were investigated utilizing Differential Scanning Calorimetry (DSC). The thermograms were measured during heating from 273 to 478 K with a rate equal to 10 K/min. Since the mixtures additionally contain water (a broad endothermic event was registered in the vicinity of 300–380 K, the result of the hygroscopic nature of the polymer), to properly examine their properties, each sample was measured with and without drying procedure. The dried samples, prior to the measurements, were annealed in the DSC device at T = 323 K for 10 min. It has been noticed that besides the disappearance of the broad thermal event reflecting the water evaporation and shift in *T_g_*, the drying procedure did not change the DSC trace of supercooled samples (see Figure 3). It should be noted that the investigated samples revealed three thermal events. First, a step-like behavior registered in the vicinity of 320–360 K that is associated with the glass transition. It is worth noting that the endotherm overshoot visible in the glass transition is associated with a physical aging phenomenon. Second, the exothermal event registered in the temperature range from 390 to 430 K, which corresponds to the sample re-crystallization. Third, the endothermal event registered at 430–360 K, reflecting the melting of bicalutamide. It must be mentioned that the presence of a single *T_g_*, coupled with the information gathered from PXRD (see Section 3.3), suggest the formation of molecular dispersions in all examined samples.

**Figure 4:**
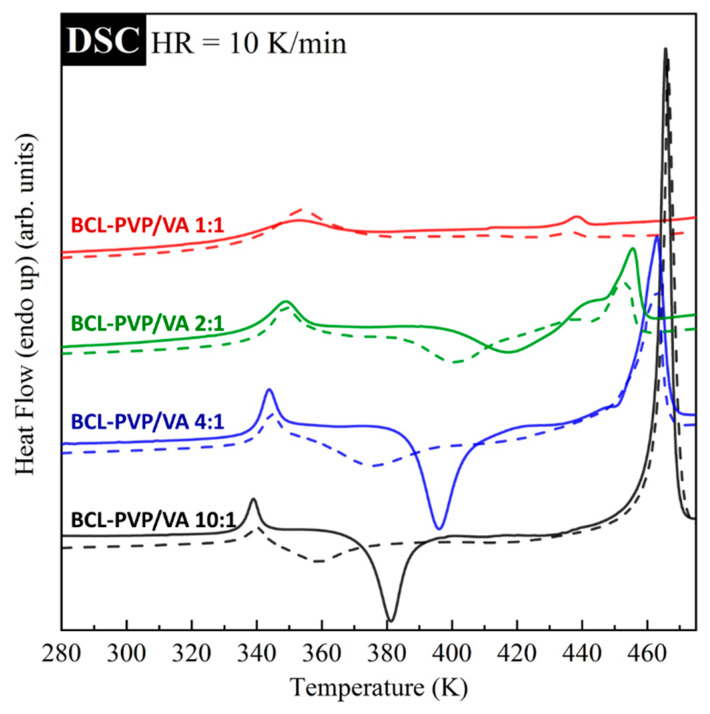
DSC thermograms of solid dispersions containing bicalutamide and KollidonVA^®^64 obtained by either spray drying (solid lines) or ball milling (dotted lines).

The effect of the amorphization method and polymer content on the thermal properties of bicalutamide is compared in Figure 4. As can be seen, in comparison to spray-dried, the milled samples are characterized by the onset of crystallization shifted towards lower temperatures. This result indicates that the samples prepared by spray drying revealed higher physical stability than milled samples. It results from the fact that the transformation in the solid phase can be incomplete. Moreover, milled samples usually exhibit lower physical stability due to the remaining nuclei or a trace amount of small crystals that are below the limits of detection of DSC or PXRD methods.

The suppression of re-crystallization was also observed when the amount of PVP/VA in solid dispersions increased. An increasing amount of the polymer leads to a higher temperature of the onset of sample crystallization, and consequently, an improved physical stability of the system. With increasing the amount of the polymer, one can also observe the increase in the glass transition temperature as well as the decrease of *T_m_*. For more clarity, the obtained values of *T_g_*, *T_c_*, and *T_m_* for all investigated systems are collected in Table 3.

**Figure 5:**
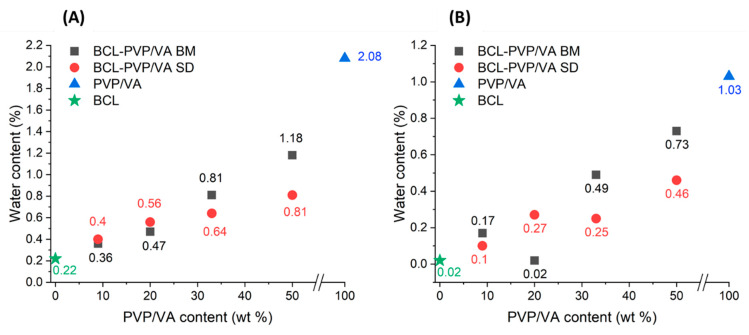
Water content in raw compounds and BCL-PVP/VA solid dispersions obtained by either ball milling (BM) or spray drying (SD) in oxidative (**A**) and the inert gas (**B**) atmosphere at 375 K.

Given the fact that PVP/VA is a hygroscopic polymer, and that water may act as a plasticizer for amorphous solids, we determined the water content in crystalline BCL, PVP/VA, and all the solid dispersions using thermogravimetric analysis (TGA). The analysis of the ‘Weight loss at 375 K’ parameter showed the same trends for both the oxidative and the inert gas atmosphere. However, the absolute values were higher in the synthetic air atmosphere (Figure 5). The highest amount of water (about 2%) under the oxidative atmosphere was lost by the reference PVP/VA copolymer. Similar behavior of PVP/VA was also revealed in the work of Patterson [27]. On the other hand, the compound with the lowest measured water content was bicalutamide, which also confirms its low water solubility [20]. Samples obtained by the ball milling method were characterized by slightly higher water content than analogous samples obtained by the spray drying method. However, regardless of the method used to obtain the samples, as the content of bicalutamide weight ratio increases, the moisture content drops. This effect can be caused by the water affinity differences between the two substances under investigation.

The effect of water was not noticeable concerning the stability of the solid dispersions. Although the amount of water was greater for samples containing a higher amount of the polymer, the *T_g_* values were the highest for these solid dispersions, indicating their stability. Similarly, the higher water content in ball-milled samples did not significantly affect their stability in comparison to spray-dried samples, as the differences in the temperatures recorded using a DSC method did not exceed 4 K.

### 3.3. Molecular Arrangement

**Figure 6:**
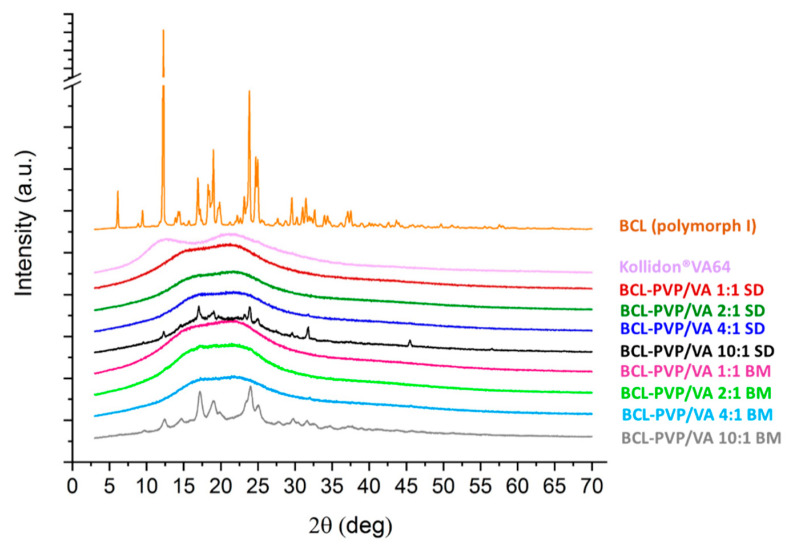
X-ray diffraction patterns of crystalline BCL and Kollidon^®^VA64 and BCL-PVP/VA solid dispersions obtained by either spray drying (SD) or ball milling (BM).

The PXRD studies were performed to characterize the molecular structure of the investigated samples. The presence of distinctive Braggs peaks indicates that bicalutamide existed as a crystal (Figure 6). The presence of peaks at 2θ equal to 6.1°, 9.4°, 12.4°, 14.4°, 17.1°–17.4°, 19.6°, 23.7°, 24.9°, 25.5°, and 31.0° respectively, corresponds to form I polymorph of monoclinic symmetry, space group P 2_1_/c, cell: a 14.882(5)Å, b 12.213(3)Å, c 10.461(3)Å, α 90°, β 104.680(13)°, γ 90°, according to the Cambridge Crystallographic Data Centre (CCDC), deposition number 602632. On the contrary, form II polymorph exhibits peaks at 2θ equal to 11.6°, 13.0°, 18.1°, 24.4°, 25.3°–25.9°, 26.7, 29.9°, and 33.6°, respectively. Although the two polymorphs have the same chemical structure, their molecular packing differs due to the differences in torsion angles [21]. It affects their physicochemical properties—form II has higher water-solubility but at the same time, this polymorph has lower physical stability.

After either spray-drying or milling with fully amorphous PVP/VA, the drug became amorphous. The destruction of the crystal lattice was manifested by an amorphous halo visible in the diffractograms of solid dispersions (Figure 6). Importantly, fully amorphous systems were obtained even when the concentration of PVP/VA in solid dispersion was as low as 20%, which confirms its high potential in the stabilization of molecularly disordered compounds. In the case of both BCL-PVP/VA 10:1 systems, there were crystalline diffraction peaks superimposed on the amorphous halos. This indicates that the samples tended to recrystallize. Although the intensities of the peaks were low, the positions of peaks at 12.4°, 17.2°, and 23.8° indicated the conversion to form I polymorph. It also confirms that the PVP/VA at a concentration below 10% is not able to sufficiently stabilize amorphous BCL. It agreed with the DSC results and SEM data, where the formation of sharp-edged particles in the spray-dried sample and crystal nuclei in ball-milled ones was noticeable.

### 3.4. Solubility Limit Studies

The recrystallization can result from the insufficient solubility of the amorphous drug in the polymer matrix. Therefore, we performed the solubility limit studies. It was recently shown that broadband dielectric spectroscopy (BDS) can be successfully utilized to determine the solubility limits of a drug dissolved within the polymer matrix [16,28,29,30,31,32]. Since the non-isothermal measurements are, without a doubt, significantly less time-consuming than isothermal ones, and at the same time they provide consistent results [16,28,29], we decided to proceed with the protocol proposed in Reference [16].

**Figure 7:**
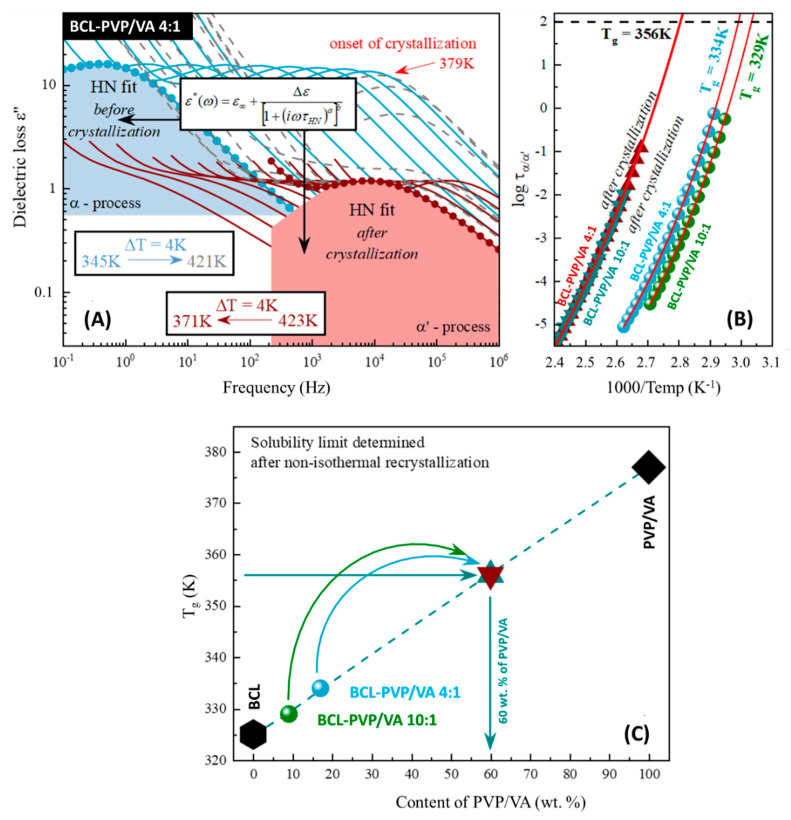
Panel (**A**) presents dielectric spectra obtained for the fully amorphous sample during non-isothermal measurements (light blue and grey lines) as well as the additional non-isothermal measurements performed after non-isothermal crystallization (maroon lines). Blue and red shaded areas correspond to the fit acquired by Havriliak–Negami (HN) function to the spectrum of fully amorphous and partially recrystallized sample presented as the light blue and maroon circles, respectively. Panel (**B**) shows fully amorphous as well as partially recrystallized BCL-PVP/VA 4:1 and BCL-PVP/VA 10:1 samples’ temperature dependency of τ_α_ and τ_α’_ in the supercooled liquid and it has been described by Vogel–Fulcher–Tamman (VFT) equations (red solid lines). Panel (**C**) displays experimentally determined concentration dependencies of the T_g_ of the BCL-PVP/VA mixtures. Maroon and turquoise triangles correspond to the concentrations obtained after the non-isothermal recrystallization.

We began our studies with the BCL-PVP/VA 4:1 system. During the non-isothermal dielectric measurements (above the sample’s *T_g_*), the α-relaxation peak—observed on the dielectric loss spectrum—shifted towards higher frequencies with increasing temperature (light blue lines in Figure 7A). By fitting the loss spectra with the Havriliak–Negami (HN) function [33], one can determine the temperature dependence of the relaxation times (*τ_α_*(T)) of the fully amorphous sample (light blue points in Figure 7B). During further heating of the sample, the excess amount of the drug started to recrystallize from the supersaturated BCL-PVP/VA 4:1 system at 379 K (see grey dashed loss spectra in Figure 7A). As the recrystallization progressed (simultaneously to further heating), one can observe the following phenomena. First, and the most expected, was the shift of the relaxation peak towards higher frequencies with the increase in the temperature. Second was the rapid decrease in the intensity of the loss peak. This sudden drop can be explained as the sample’s recrystallization since dielectric strength (*Δε*) is proportional to the number of units involved in the structural relaxation [34,35]. The last observed phenomenon was the most complex one as it was partially compensated by the first one mentioned above. It must be pointed out that the used polymer matrix has higher glass transition temperature than amorphous bicalutamide and therefore, one can observe the anti-plasticization effect exerted by its addition (deceleration of the drug’s molecular mobility).

Along with the recrystallization of the excess amount of the drug from the supersaturated mixture, the changes in the amorphous drug-polymer ratio can be observed (apparent increase in the amount of polymer). The effect of this change is an increase in the anti-plasticization effect (further deceleration of the drug’s molecular mobility), which will be manifested as the shift of the relaxation peak towards lower frequencies. When the drug-polymer ratio does not change anymore, a decrease in the intensity of the loss peak can be observed (see grey dashed line in Figure 7A). The crystallization process was terminated—this suggested that the excess amount of the drug recrystallized, and the resulted amount did not display any tendency towards recrystallization during the non-isothermal studies.

Then, the sample was remeasured again (see maroon line in Figure 7A), to cover the wide temperature range of the *τ_α_’*(T) (see maroon triangles in Figure 7B). Temperature evolution of the structural relaxation time—in the supercooled liquid region—usually shows non-Arrhenius-like behavior. Hence, to parameterize it, we used the Vogel–Fulcher–Tamman (VFT) equation [36,37,38], that is defined as follows:

(1) $$\tau_{\alpha }\left(T\right)=\tau_{\infty }\exp\left(\frac{B}{T-T_{0}}\right)$$

where *τ_∞_*, *B*, and *T_0_* are the fitting parameters.

To determine the glass transition temperature related to the α– and α’–process, we extrapolated the VFT fit to 100 s (*T_g_* = *T*(*τ_α_* = 100 s)). Thus, the fully amorphous BCL-PVP/VA 4:1 system was characterized by a glass transition temperature equal to 334 K (which was in good agreement with the calorimetric data, considering differences between heating rates applied during dielectric and calorimetric measurements), while the *T_g_* of the system obtained after non-isothermal crystallization was equal to 356 K (see Figure 7B). The whole procedure was repeated for different initial concentration (BCL-PVP/VA 10:1) and obtained results led us to the same, stable concentration (see Figure 7B).

Once the *T_g_* value obtained after recrystallization was confirmed, it was possible to determine the concentrations of the corresponding mixture, by comparing this result to the experimentally determined (via BDS) concentrations dependency of the glass transition temperature of BCL-PVP/VA systems (see Figure 7C). Accordingly, one can observe that the solubility limits of the BCL within the PVP/VA matrix determined during the non-isothermal measurements is equal to 40 wt.%.

### 3.5. Infrared Spectroscopy (FTIR)

The effect of stabilization of amorphous APIs by polymers can be attributed to either intermolecular interactions between the components of solid dispersions or the anti-plasticizing effect directly connected with the glass transition temperature [39]. FTIR spectroscopy allows to identify the structure and the interactions occurring between molecules as well as to distinguish the crystalline and amorphous form of drugs based on the differences between peak width, intensity, shape, and position.

**Figure 8:**
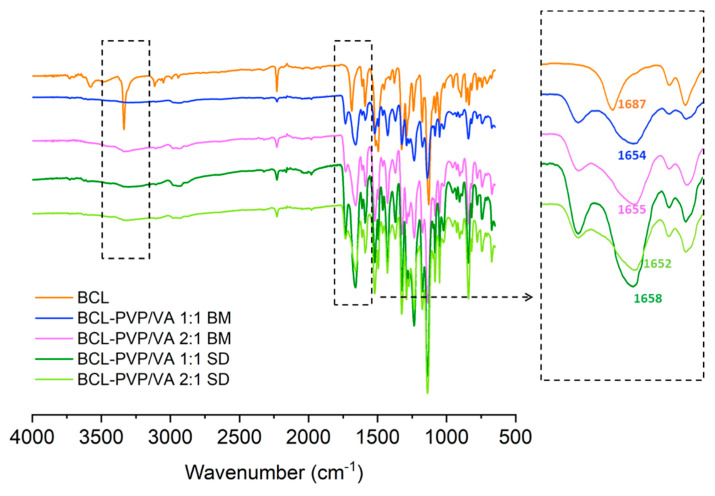
Fourier Transform Infrared (FTIR) spectra of crystalline BCL and selected solid dispersions (BM—ball milling, SD—spray drying).

The spectrum of crystalline BCL is characterized by intense peaks of stretching vibrations of functional groups such as carbonyl, amide, nitrile, hydroxyl, and sulfonyl (Figure 8). Given the molecular structure of the BCL molecule, the presence of proton acceptors such as carbonyl, sulfonyl, or nitrile group, and hydrogen atom donors, i.e., hydroxyl and amide group, it is supposed to form hydrogen bonds with other molecules. The spectra of solid dispersions indicated that BCL interacts with PVP/VA, which is particularly noticeable in the region characteristic for the vibrations of the amide bond. The redshift of the C=O absorption band from 1687 cm^−1^ registered for crystalline BCL to 1652–1658 cm^−1^ for solid dispersions confirmed that drug molecules interacted with the polymer. Moreover, the band of BCL at 3336 cm^−1^ assigned to N-H stretching vibrations disappeared, which indicated the tautomeric conversion from the amide form to a less stable imidic one [40]. Those intermolecular interactions were responsible for the observed potential of PVP/VA to stabilize amorphous bicalutamide.

### 3.6. The Effect of Compression on Molecular Arrangement

Bicalutamide is a compound known for its propensity to undergo mechanical activation [23,41]. In a mechanical process such as milling, the substance subjected to a mechanical treatment gains the energy that must be released. The relaxation of the stress field can occur in several ways, including thermal decomposition, accumulation of defects in the crystal structure, formation of new interfaces or metastable polymorphs, and amorphization. It can also result in a chemical reaction, depending on the thermal and mechanical properties of the solid material as well as the mechanical treatment rate [42]. On the other hand, amorphous bicalutamide is known to recrystallize easily upon mechanical treatment, such as crushing or grinding in a mortar [43]. The addition of PVP has been described to hinder the propensity towards recrystallization and improve the physical stability of solid dispersions [44].

While the effect of prolonged mechanical treatment during milling was described for bicalutamide, there have been no investigations on the effect of compression published so far. It raises two questions: how the compression affects the crystalline drug and what happens when the amorphous systems are subjected to increased pressure. In our studies, we compressed crystalline bicalutamide (form I polymorph) physically mixed with PVP/VA as well as solid dispersions obtained in either milling or spray drying processes. We aimed at checking how the applied force affects the molecular structure of the drug. It is an important issue from the perspective of pharmaceutical technology as the physical stability of active compounds needs to be ensured during the manufacturing and storage of the dosage form. Polymorph transitions and amorphization-recrystallization events can alter the dissolution, and thus the absorption of the drug.

**Figure 9:**
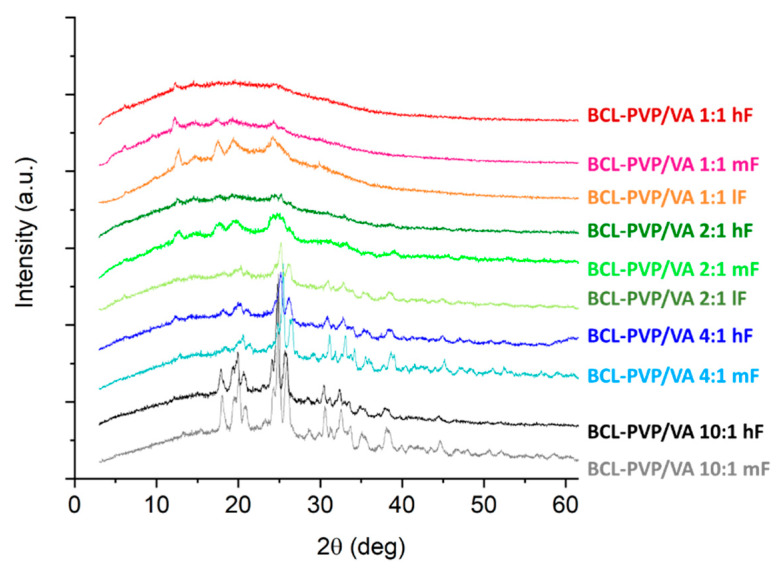
X-ray diffraction patterns of tablets containing BCL physically mixed with PVP/VA (hF, mF, and lF represent high, medium, or low compression force, respectively).

The performed PXRD experiments revealed that the compression led to the amorphization of crystalline bicalutamide physically mixed with the polymer (Figure 9). The collected diffraction patterns indicated the disruption of the crystal lattice. The system containing 50% of the drug compressed at 4.83 kN was amorphous, while those compressed with lower forces remained partially crystalline, as confirmed by the presence of Braggs peaks superimposed on the amorphous halo. The higher the content of the drug substance in the mixtures, the more pronounced the Braggs peaks. Interestingly, the compression induced a transition from the form I to form II polymorph of bicalutamide, as indicated by the characteristic peak at 2θ = 25.9°.

It confirmed the high susceptibility of bicalutamide to undergo polymorph transition and amorphization upon even short-term mechanical stress and stabilizing properties of PVP/VA. However, polymeric excipient was found to stabilize molecularly disordered bicalutamide only when its amount was equal to drug content and the compression force was sufficiently high.

The development of tablets containing amorphous solid dispersions can serve as a strategy to improve drug dissolution characteristics, and thus its bioavailability. However, the stability still acts as one of the biggest limitations. The recrystallization can undermine the beneficial effects of amorphous compounds. It can also cause severe effects because it affects the pharmacokinetic profile of the drug. Thus, the investigations of the molecular structure of the drug compound during every step of the drug development process must be performed.

**Figure 10:**
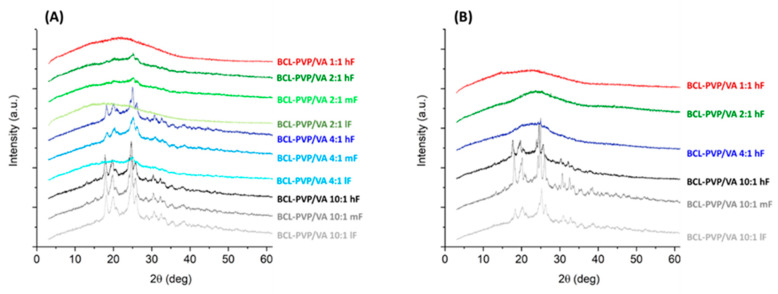
X-ray diffraction patterns of tablets containing BCL-PVP/VA solid dispersions obtained by either ball milling (**A**) or spray drying (**B**). hF, mF, and lF represent high, medium, or low compression force, respectively.

We examined the effect of compression on the molecular arrangement of bicalutamide in amorphous solid dispersions. The PXRD results confirmed that the compression induced the recrystallization of bicalutamide. With increasing compression force, a propensity towards the recrystallization increases. However, the effect differed regarding both the content of PVP/VA in the system and the method of amorphization. The diffraction patterns collected for both BCL-PVP/VA 1:1 systems indicated that the polymer stabilized the amorphous drug since no evidence for drug recrystallization was recorded after applying the highest compression force (Figure 10). With the decrease in polymer content, the differences between systems obtained using different amorphization methods became significant. Spray-dried solid dispersions containing 33% and 20% of the polymer remained amorphous after compression with 5 kN force (Figure 10B). The milled samples recrystallized upon either medium compression force, i.e., 2.3 kN for BCL-PVP/VA 2:1 system or low compression force, i.e., 0.24 kN for BCL-PVP/VA 4:1 solid dispersion (Figure 10A). It agrees with the results of thermal analysis indicating the higher stability of spray-dried systems given by the higher temperature of the onset of sample crystallization.

Regardless of the amorphization method, the systems containing 90% of BCL exhibited the most noticeable Braggs peaks. Interestingly, compression led to the conversion to form II polymorph, as indicated by the position of diffraction peaks, especially the one at ca. 25.9°, which is characteristic for the second polymorph.

The obtained results indicate that the development of tablets containing amorphous solid dispersions with bicalutamide requires a great amount of stabilizing polymer. Given the binding properties of PVP/VA, it may lead to retarded drug dissolution and difficult tablet disintegration.

### 3.7. Dissolution of Bicalutamide

The amorphization is a commonly used technique applied to enhance the dissolution of poorly water-soluble drugs. Due to the limited stability of the molecularly disordered systems, the addition of great excess of the polymer is usually required. Such a high polymer concentration ensures rapid dissolution of the matrix and release of very fine drug particles having very high surface area, which results in dissolution rate enhancement. It was described by Andrews et al. [45], who applied hot-melt extrusion to obtain BCL-PVP solid dispersions containing 77%–91% of the carrier. The authors assigned the physical stability of extrudates to the inhibitory effect of PVP on BCL crystallization. The dissolution was enhanced by 7.5- to 8.9-fold, as compared with the crystalline drug [45].

**Figure 11:**
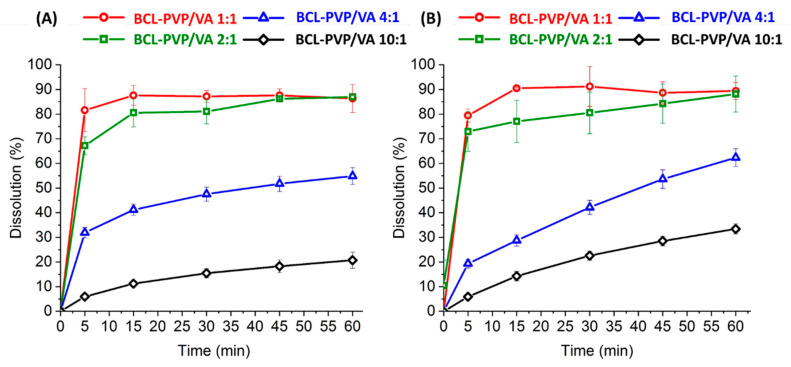
Dissolution profiles of bicalutamide from solid dispersions obtained by either ball milling (**A**) or spray drying (**B**).

Our study aimed at using low concentrations of the polymer as it allows for maintaining the necessary dose of API in a small-weight dosage form. We observed that the increase in the amount of dissolved bicalutamide follows the increase of the content of PVP/VA. However, no differences were noticed when the polymer concentration exceeded 33%. After one hour, (20.7 ± 3.3)% of BCL dissolved from the 10:1 milled system, (54.9 ± 3.5)% from the 4:1 solid dispersion, (86.9 ± 1.1)% from the 2:1 system, and 86.4% ± 5.7% from the 1:1 composition (Figure 11A). The profiles of spray-dried solid dispersions follow the same trend, with (33.4 ± 2.1)%, (62.4 ± 3.6)%, (88.1 ± 7.3)%, and (89.4 ± 3.4)% of the drug dissolved from BCL-PVP/VA 10:1, 4:1, 2:1, and 1:1 solid dispersions, respectively (Figure 11B). Given that only (8.2 ± 1.0)% of crystalline bicalutamide dissolved after one hour of dissolution test, the relative enhancement of drug dissolution varied from 2.5 to 11 times.

We performed the dissolution studies using an intrinsic dissolution set to verify if the improvement in the dissolution rate is the matter of increased surface area. In the intrinsic dissolution rate (IDR) studies, the powder is compressed with high force. It prevents the particle size or porosity of the sample from affecting the dissolution. The issue of the powder floating on the surface of the dissolution medium is also eliminated.

From the data presented in Table 4, it can be concluded that the formulations prepared by ball milling are characterized by a faster dissolution rate. It is well pronounced, especially for the formulations with a higher amount of the polymer, i.e., 1 to 1 and 2 to 1 ratio. Given the results from particle size measurements, the dissolution from powder systems, and intrinsic dissolution rate determination, it can be concluded that the impact of the larger particle size of the ball-milled samples was minimized by its higher intrinsic dissolution rates. Thus, the bicalutamide dissolution profiles from corresponding formulations prepared either by ball milling or spray drying are comparable. Significantly slower dissolution from BCL-PVP/VA formulation prepared by spray drying in the first 15 min of the dissolution test can be attributed to floating properties of the powder on the surface of the dissolution medium, while powder prepared by ball milling sank immediately due to the higher density. Nevertheless, the IDR results revealed that ball milling produced powder systems characterized by faster bicalutamide dissolution.

### 3.8. Wettability Study

Given that the wetting of a solid surface is the first step in the dissolution process, we applied a sessile drop technique to investigate the water contact angles. They reflect the hydrophobicity of the surface of the considered system, which means that they are a measure of the wettability of compressed solid dispersions by water [46]. The low water solubility of bicalutamide is correlated with the hydrophobicity of the compound. The value of the contact angle of crystalline BCL is equal to 74.13°, which indicates that the surface is only slightly wet by water and the contact with water molecules is limited. The formation of solid dispersions with a hydrophilic PVP/VA polymer described by a contact angle equal to 43.16° led to an increase in wettability given as a decrease in the values of measured contact angles (Table 5).

**Figure 12:**
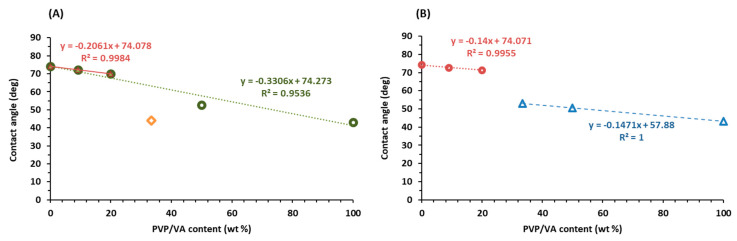
The values of contact angle of solid dispersions obtained by either ball milling (**A**) or spray drying (**B**).

The values are close to 50° for the systems containing 50% and 66% of BCL, and 70° for the systems containing more than 80% of the drug, regardless of the solid dispersion preparation method. Figure 12 shows the values of contact angles measured for pure compounds and binary solid dispersions. Interestingly, the values of contact angle were almost a linear combination of the wettability of pure drug and polymer that the tablet is composed of. It suggested that the PVP/VA-based solid dispersions presented surface properties which did not influence the interactions of each other with water. In other words, the intermolecular interaction between the components did not lead to a reorganization of the surface groups for either of the components [47]. However, in the case of spray-dried systems, the two regions of linearity can be distinguished, i.e., first for systems containing less than 20% of PVP/VA, and second for solid dispersions composed of more than 30% of this polymer (Figure 12B).

For ball-milled systems, the inflection point is also located at around 30%-content of PVP/VA; however, after elimination of this point as an outlier, the values of contact angle are linearly correlated with the PVP/VA content in the whole concentration range (Figure 12A). Similarly, the R^2^ value of the linear fitting adjusted to the first three points of Figure 12A (milled samples) is almost equal to unity. The results indicated that hydrophobicity of solid dispersions can be affected differently by the various quantities of the hydrophilic polymer [48].

The increase in wettability corresponds to the dissolution data as more drug dissolved from solid dispersions characterized by lower values of contact angle, i.e., systems containing a higher amount of polymer. The retarded dissolution of bicalutamide from binary mixtures of high-drug content resulted from the lower concentration of hydrophobic molecules in fine particulate form. The hydrophobic nature of the drug-rich diffusion layer leads to a more difficult wetting of the surface and thus becomes a rate-limiting step in the dissolution process [49,50].

## 4. Conclusions

The performed studies indicated that both applied methods, i.e., ball milling and spray drying, led to amorphization of bicalutamide. The transfer of mechanical energy led to clearly marked changes in particle morphology, as no sign of neither hexagonal crystals of bicalutamide or the spheres of Kollidon^®^VA64 was observed. The irregular particles of rough surface and more spherical shape were present instead. The spray drying process led to the formation of spherical particles not exceeding 10 µm in diameter.

The obtained amorphous solid dispersions were not susceptible to recrystallize, as confirmed by XRD and DSC studies. It was a result of intermolecular interactions of hydrogen bonding type. It was confirmed that an increase in polymer content is correlated with the lower propensity towards recrystallization of solid dispersion, regardless of the applied process. It was correlated with the high miscibility between the two components—the non-isothermal dielectric investigations revealed that 40% of BCL dissolves in PVP/VA. Moreover, the results indicated that spray-dried systems exhibit better stability as the onset of crystallization temperature shifts towards higher values.

The observed particle size reduction, phase transition of bicalutamide, and the increase in wettability resulted in the 2.5- to 11-fold improvement of drug dissolution from solid dispersions.

Short-term mechanical force applied during compression of BCL-PVP/VA physical mixtures led to drug amorphization. With increasing content of the drug in the sample, the diffractograms were more structured, which indicated drug crystallization. Interestingly, the compression-induced conversion from form I to form II polymorph occurred. Given the propensity of high-energy solids towards crystallizations, we tested the effect of compression on molecular arrangement of bicalutamide in solid dispersions. The results indicated that the drug remained amorphous when the content of PVP/VA exceeded 20% or 33%, in the case of spray-dried and milled systems, respectively. It resulted from lower propensity towards recrystallization of spray-dried solid dispersions. In the case of both BCL-PVP/VA 1:10 systems, the conversion to form II polymorph was confirmed.

Given the described phase transition, the obtained results indicate that the properties of the molecular system need to be carefully considered during the dosage form development.

## Figures and Tables

**Table 1:** Compression force ranges used during tableting.

Level of compression force	Force range (kN)	Pressure range (MPa)
low (lF)	below 0.92	below 23.92
medium (mF)	1.42–1.78 *	36.92–46.28
high (hF)	4.69–5.37	121.93–139.61

* Two systems assigned to the medium compression range group were compressed using higher forces, 2.39 kN and 3.14 kN, to obtain tablets. They were: BCL-PVP/VA 2:1 spray-dried system and BCL-PVP/VA 10:1 physical mixture, respectively.

**Table 2:** Particle size of solid dispersions obtained using the laser diffraction method.

BCL-PVP/VA wt. ratio	Type of process	D_v_(50) ± SD (µm)	Span^a^
1 to 1	Ball milling	50.7 ± 9.5	5.50
2 to 1		43.5 ± 7.5	5.41
4 to 1		109.6 ± 12.1	5.93
10 to 1		105.0 ± 4.7	5.07
1 to 1	Spray drying	20.3 ± 1.7	1.72
2 to 1		23.0 ± 1.8	4.54
4 to 1		32.3 ± 0.5	1.78
10 to 1		20.2 ± 0.1	3.59

^a^*Span = {D_v_(*90*) − D_v_(*10*)}/D_v_(*50*)*, where *D_v_*(10), *D_v_*(50), and *D_v_*(90) represent the size of 10%, 50%, and 90% of the total volume of material in the sample, respectively.

**Table 3:** Comparison of the T_g_, T_c_, and T_m_ values of solid dispersions containing bicalutamide and Kollidon^®^VA64 obtained by either ball milling or spray drying.

BCL-PVP/VA wt. ratio	Type of process	T_g_ (K)	T_c_ (K)	T_m_ (K)
1 to 1	Ball milling	341	414	437
2 to 1		340	385	453
4 to 1		337	359	463
10 to 1		333	344	466
1 to 1	Spray drying	339	418	438
2 to 1		338	398	455
4 to 1		336	387	463
10 to 1		331	374	466

**Table 4:** The intrinsic dissolution rates obtained for prepared formulations.

BCL-PVP/VA wt. ratio	Type of process	IDR (mg/cm^2^/min)	SD
1 to 1	Ball milling	2.034	0.012
2 to 1		1.808	0.083
4 to 1		0.034	0.003
10 to 1		0.028	0.004
1 to 1	Spray drying	0.989	0.007
2 to 1		0.177	0.021
4 to 1		0.031	0.005
10 to 1		0.021	0.003

**Table 5:** The values of contact angle of solid dispersions obtained using the sessile drop technique.

	BCL-PVP/VA ratio	Contact angle (degree)			
Process		1:1	2:1	4:1	10:1
**Ball milling**		52.75	44.00	70.00	72.11
**Spray drying**		50.56	52.95	71.32	72.69
